# Evidence of Rickettsia helvetica infection in humans, eastern France.

**DOI:** 10.3201/eid0604.000412

**Published:** 2000

**Authors:** P E Fournier, F Grunnenberger, B Jaulhac, G Gastinger, D Raoult

**Affiliations:** Université de la Méditerranée, Marseille, France.

## Abstract

A 37-year-old man living in eastern France seroconverted to Rickettsia helvetica in August 1997, 4 weeks after the onset of an unexplained febrile illness. Results of a serosurvey of forest workers from the area where the patient lived showed a 9.2% seroprevalence against R. helvetica. This organism may pose a threat for populations exposed to Ixodes ricinus ticks.


### Evidence of *Rickettsia helvetica* Infection in Humans, Eastern France

**Pierre-Edouard Fournier,* Fabienne Grunnenberger,† Benoît Jaulhac,‡ Geneviève Gastinger,§ and Didier Raoult***

*Université de la Méditerranée, Marseille, France; †Hopital de Hautepierre, Strasbourg, France; ‡Faculté de Médecine, Strasbourg, France; and §Mutualité Sociale Agricole du Bas-Rhin, Strasbourg, France

A 37-year-old man living in eastern France seroconverted to *Rickettsia helvetica* in August 1997, 4 weeks after the onset of an unexplained febrile illness. Results of a serosurvey of forest workers from the area where the patient lived showed a 9.2% seroprevalence against *R. helvetica*. This organism may pose a threat for populations exposed to *Ixodes ricinus* ticks.

Spotted fever group rickettsiae are gram-negative intracellular bacilli associated with arthropods and transmitted by ticks. The most common clinical features of rickettsioses in humans are fever, headache, rash, and inoculation eschar. Five well-characterized and three recently proposed rickettsioses of humans have been identified in the past 13 years. Extensively studied rickettsioses include Japanese spotted fever (caused by *Rickettsia japonica*), Astrakhan fever (Astrakhan fever rickettsia), Flinder's Island spotted fever (*R. honei)*, California flea typhus (*R. felis)*, and African tick-bite fever (*R. africae)*. In France, *R. conorii*, the agent of Mediterranean spotted fever, which is transmitted through the bite of the brown dog tick *(Rhipicephalus sanguineus*), is the main pathogenic rickettsia and is encountered in the southern part of the country. Recently, *"Rickettsia mongolotimonae,"* *R. slovaca*, and *R. helvetica* have also been identified as agents of human rickettsioses in Europe. Other species of unknown pathogenicity—including *R. rhipicephali*, *R. massiliae*, and Bar 29—have been isolated from ticks in Europe.

*R. helvetica* has been isolated from *Ixodes ricinus* collected in Switzerland, France, Slovenia, and Sweden. These ticks, which readily bite humans, are also the vectors in Europe of *Borrelia burgdorferi* and *Ehrlichia phagocytophila*, the agent of human granulocytic ehrlichiosis. *I. ricinus* is widely prevalent in parts of Europe (1), including eastern France, and frequently bites humans (Figure 1). Consequently, the potential transmission of *R. helvetica* by *I. ricinus* to humans seemed likely, but its pathogenic capacity in this regard remained uncertain until Nilsson et al. demonstrated its role in the development of perimyocarditis and sudden death in two young patients (2).

In this report, we describe a human infectious syndrome in which specific seroconversion against *R. helvetica* occurred. We also present the results of a serosurvey on rickettsioses conducted in Alsace (eastern France) among forest workers,

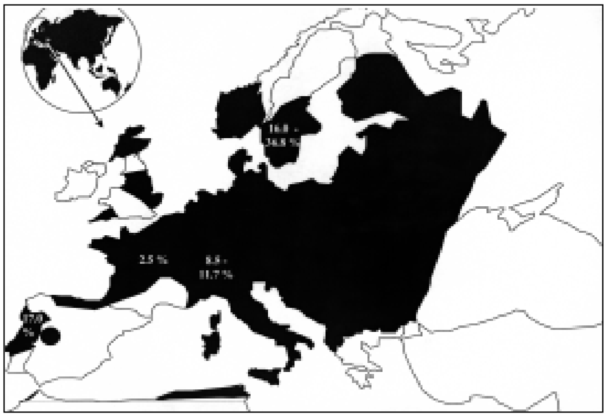

Figure 1. Map of Europe showing the distribution of *Ixodes ricinus* (1). The areas where *I. ricinus* is prevalent are shaded in black and the prevalence of *R. helvetica* in *I. ricinus*, where estimated, is indicated.

Address for correspondence: Didier Raoult, Unité des Rickettsies, CNRS: UPRESA 6020, Faculté de Médecine, Université de la Méditerranée, 27, Boulevard Jean Moulin, 13385 Marseille cedex 05, France; fax: 33-04-91-83-0390; e-mail: Didier.Raoult@medecine.univ-mrs.fr.

which showed high prevalence of antibody reactive with this bacterium.

### Case Report

A 37-year-old, immunocompetent man was admitted to a hospital in Strasbourg, Alsace, on August 18, 1997, with prolonged fever, fatigue, myalgias, and headache of unknown etiology. The fever had begun on August l, 18 days after the man walked in a forest near Strasbourg, where he observed numerous ticks but not any tick bites. On physical examination, the physician observed low-grade fever (38.1°C) but no rash, lymphadenopathy or inoculation eschar.

Results of laboratory tests included an elevated C-reactive protein (48 mg/L); increased fibrinogen level (6.0 g/L); accelerated erythrocyte sedimentation rate (34 mm, first hour); leukocyte count of 6,700/mm$^3$; platelet count of 165,000/mm$^3$; and increased rates of alanine-aminotransferase (60 IU/L) and aspartate aminotransferase (52 IU/L). Blood cultures taken on August 18 were negative. The patient received no antibiotic treatment and recovered spontaneously within 2 weeks. Rickettsial serologic tests were performed on serum samples (taken on days 17, 27, and 86 after onset) at the Unité des Rickettsies. Antibody levels against *B. burgdorferi*, *Francisella tularensis*, *E. phagocytophila*, and hepatitis A, B and C viruses were measured in Strasbourg but were negative.

### Serosurvey

A convenience collection of serum samples from 379 forest workers from Alsace, all of whom were state employees, was obtained. These samples had been initially collected as part of a systematic serosurvey organized by the state department of occupational medicine to evaluate for the presence of antibodies to *B. burgdorferi*. This population was composed of 377 men and 2 women, 20 to 59 years of age; 360 (95.5%) reported frequent tick bites but were clinically asymptomatic; the remaining 19 reported no tick bites. No questionnaire was administered. After informed consent from the patients, antibodies to *R. helvetica*, *R. conorii*, *R. slovaca,* and *"R. mongolotimonae"* were determined.

### Serologic Tests

To determine the specificity of cross-reactions, serum samples, including those from the survey, were adsorbed with *R. conorii*, *R. helvetica*, *R. slovaca* or *R. mongolotimonae* antigens. Cross-adsorption procedures were performed (3). *R. helvetica*, *R. conorii*, *R. slovaca*, and "*R. mongolotimonae*" Western blot analyses were performed on the resultant supernatants. Purified *R. helvetica*, *R. conorii*, *R. slovaca*, and "*R. mongolotimonae*" were suspended in sterile water and adjusted to 2 mg/mL with a UV spectrophotometer. Western blotting procedures using unheated antigens were performed (4). Antibodies reacting against antigens of 20 to 50 kDa were assumed to be directed primarily against the lipopolysaccharide in *R. helvetica*, *R. conorii*, *R. slovaca*, and "*R. mongolotimonae.*" Serologic specificity toward the different spotted fever group rickettsia species was determined by the relative immunoglobulin G (IgG) reactivity to species-specific antigens in the 110- to 145-kDa region. Intensity measures were compared by video image acquisition (The Imager, Appligene, Illkirch, France).

### Case Findings

The first serum sample, taken from the patient on August 18, was negative for all tested antigens. Samples taken on August 27 and October 27 yielded IgG titers of 1:128, 1:64, 1:128 and 1:256, and IgM titers of 1:64, 1:16, 1:32 and 1:128 against *R. conorii*, *R. slovaca*, "*R. mongolotimonae,*" and *R. helvetica*, respectively, but were negative for *Coxiella burnetii* and *E. phagocytophila*. Western immunoblotting of the patient's serum samples demonstrated cross-reactivity between *R. helvetica*, *R. slovaca*, "*R. mongolotimonae,*" and *R. conorii* antigens, but adsorption with *R. helvetica* antigen eliminated all antibodies. After adsorption with *R. conorii*, *R. slovaca*, and "*R. mongolotimonae*" antigens, antibodies to *R. helvetica* were still present, but antibodies to the other three antigens were not observed, indicating that *R. helvetica* was the etiologic agent.

### Serosurvey Findings

Of the 379 patients included in our serosurvey, 35 (9.2%) demonstrated anti-*R. helvetica* IgG titers 1:64 (16 had an IgG titer of 1:64; 13 a titer of 1:128; 5 a titer of 1:256; and one a titer of 1:512). IgM was not detected in any patient. In all positive serum samples, titers were greater against *R. helvetica* than against any of the other three tested antigens by two or more dilutions (Figure 1). Following adsorption with

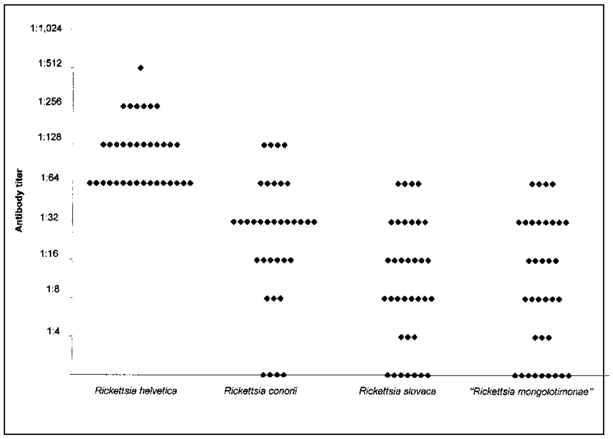

Figure 2. Antibody titers against *Rickettsia helvetica*, *R. conorii*, *R. slovaca*, and "*R. mongolotimonae*" in 35 forest workers, Alsace.*

*Each diamond represents the IgG titer in serum samples from the patients. *R. conorii* Moroccan strain (ATCC VR 141), *R. helvetica* C9P9 strain, *R. slovaca* 13-B strain, and "*R. mongolotimonae*" HA91 strain were grown on confluent layers of Vero cells in a 150-cm$^2$ flask. Infection was monitored by Gimenez staining. When 90% of the cells were infected, cell layers and supernatants were harvested, pelleted by centrifugation (10,000 x *g* for 10 min), and resuspended in 15 mL of phosphate-buffered saline (PBS, pH 7.3). Antigen purification was performed (5). The final suspension was resuspended in sterile water, and the protein content of the purified organisms was determined by UV spectrophotometry and adjusted to 1 mg/mL. All four rickettsial antigens were applied by pen point to each well of 30-well microscope slides (Dynatech Laboratories Ltd., Billingshurt, United Kingdom), air dried, and fixed with acetone for 10 min. This allowed a direct comparison of fluorescence intensity between antigens. All serum samples, including positive and negative controls, were diluted 1/4, 1/8, 1/16, 1/32, 1/64 and 1/128 in PBS with 3% nonfat dry milk. Microimmunofluorescence procedures were performed (3). All serum samples found to be positive for total immunoglobulins were diluted serially (twofold dilutions ranging from 1/32 to 1/2,048 or more), and the titers of IgG and IgM were determined. Before detection of IgM, serum was adsorbed with a rheumatoid factor absorbent (RF-absorbent, Behring-werke AG, Marburg, Germany). Titers at or above 1:64 for IgG or 1:32 for IgM were considered positive.

*R. helvetica* antigen, all antibodies were eliminated.

### Conclusions

Epidemiologic evidence suggests that our patient was exposed to *I. ricinus* during his forest walk. He did not report any tick bites, but larvae or nymphs are often not detected by patients. Clinically, he presented with a prolonged low-grade fever, headache and myalgias. Unfortunately, no blood specimen was available for culture. Though neither cardiac symptoms nor a rash developed, the latter is sometimes lacking in other spotted fever group rickettsioses, such as African tick-bite fever or *R. slovaca* infection.

The usual method for the diagnosis of rickettsioses is serologic testing; the easiest method is microimmunofluorescence. Although serological cross-reactions are common among spotted fever group rickettsiae (6), *R. helvetica* is phylogenetically distant from other spotted fever group rickettsiae as well as from typhus group rickettsiae. Thus, one could expect the antibody response to this bacterium to be higher than against other spotted fever group rickettsiae. In our patient and 35 (9.2%) of 379 tested forest workers, antibody titers against *R. helvetica* were higher than those detected against *R. conorii*, *R. slovaca*, and *"R. mongolotimonae."* The specificity of the antibody response was confirmed by cross-adsorption and Western blotting. The forest workers lived in Alsace, an area where *R. conorii* and *Rhipicephalus* sp. are absent but where *I. ricinus* is highly prevalent, and were frequently exposed to ticks. To date, *I. ricinus* has only been demonstrated to harbor one rickettsial species, *R. helvetica*. Therefore, the observed seroprevalence was mainly or fully caused by *R. helvetica*. Although none of the forestry workers in our study reported any tick-bite-related disease, such results suggest that this occupational group may be exposed to the bites of *I. ricinus* and to *R. helvetica* infection. Prospective evaluation of ill patients and molecular identification of the causative agents need to be conducted.

Our data support the notions that this rickettsia, found in ticks (*I. ricinus)* that bite humans and are widely distributed in forest areas in Central Europe, could be frequent and that *I. ricinus* should be considered in the diagnosis of tick-transmitted infection in Europe.

Dr. Fournier is a physician in the French reference center for the diagnosis and study of rickettsial diseases. His research interests include the clinical and epidemiologic features of rickettsioses.

### References

1. Gilot B. Bases biologiques, écologiques et cartographiques pour l'étude des maladies transmises par les tiques (Ixodidae et Argasidae) dans les alpes françaises et leur avant pays) [thesis]. Grenoble, France: Universite de Grenoble]; 1985.

2. Nilsson K, Lindquist O, Pahlson C. Association of *Rickettsia helvetica* with chronic perimyocarditis in sudden cardiac death. Lancet 1999;354:1169-73.
3. Brouqui P, Harle JR, Delmont J, Frances C, Weiller PJ, Raoult D. African tick bite fever : an imported spotless rickettsiosis. Arch Intern Med 1997;157:119-24.
4. Teysseire N, Raoult D. Comparison of Western immunoblotting and microimmunofluoresence for diagnosis of Mediterranean spotted fever. J Clin Microbiol 1992;30 :455-60.
5. Raoult D, Dasch GA. Line blot and Western blot immunoassays for diagnosis of Mediterranean spotted fever. J Clin Microbiol 1989;27:2073-9..
6. Hechemy KE, Raoult D, Fox J, Han Y, Elliott LB, Rawlings J. Cross-reaction of immune sera from patients with rickettsial diseases. J Med Microbiol 1989;29:199-202.
